# Genome-Wide Association Study and Fine Mapping Reveals Candidate Genes for Birth Weight of Yorkshire and Landrace Pigs

**DOI:** 10.3389/fgene.2020.00183

**Published:** 2020-03-27

**Authors:** Yong Li, Bin Li, Manman Yang, Hu Han, Tao Chen, Qiang Wei, Zepu Miao, Lilin Yin, Ran Wang, Junran Shen, Xinyun Li, Xuewen Xu, Ming Fang, Shuhong Zhao

**Affiliations:** ^1^Key Laboratory of Agricultural Animal Genetics, Breeding and Reproduction, The Cooperative Innovation Center for Sustainable Pig Production, Ministry of Education, Huazhong Agricultural University, Wuhan, China; ^2^Shenzhen Engineering Laboratory for Genomics – Assisted Animal Breeding, BGI Institute of Applied Agriculture, BGI-Shenzhen, Shenzhen, China; ^3^Key Laboratory of Healthy Mariculture for the East China Sea, Ministry of Agriculture and Rural Affairs, Fisheries College, Jimei University, Xiamen, China

**Keywords:** birth weight, fine mapping, candidate genes, GWAS, pig

## Abstract

Birth weight of pigs is an important economic factor in the livestock industry. The identification of the genes and variants that underlie birth weight is of great importance. In this study, we integrated two genotyping methods, single nucleotide polymorphism (SNP) chip analysis and restriction site associated DNA sequencing (RAD-seq) to genotype genome-wide SNPs. In total, 45,175 and 139,634 SNPs were detected with the SNP chip and RAD-seq, respectively. The genome-wide association study (GWAS) of the combined SNP panels identified two significant loci located at chr1: 97,745,041 and chr4: 112,031,589, that explained 6.36% and 4.25% of the phenotypic variance respectively. To reduce interval containing causal variants, we imputed sequence-level SNPs in the GWAS identified regions and fine-mapped the causative variants into two narrower genomic intervals: a ∼100 kb interval containing 71 SNPs and a broader ∼870 kb interval with 432 SNPs. This fine-mapping highlighted four promising candidate genes, *SKOR2*, *SMAD2*, *VAV3*, and *NTNG1*. Additionally, the functional genes, *SLC25A24*, *PRMT6* and *STXBP3*, are also located near the fine-mapping region. These results suggest that these candidate genes may have contribute substantially to the birth weight of pigs.

## Introduction

The birth weight of pigs is an important economic trait in the livestock industry. It is closely associated with early survival, weaning weight, and growth rate after weaning (Quiniou et al., 2002; Smith et al., 2007). Pigs have been selectively bred to produce larger litters, however, with this increase in litter size, the average birth weight has decreased (Bergstrom et al., 2009; De Almeida et al., 2014). Birth weight reflects the intrauterine growth of piglets which is affected by both the maternal supply of nutrition and genetic factors (Roehe, 1999; Zohdi et al., 2012). Measures of birth weight heritability have ranged from 0.08 to 0.36 (Roehe, 1999; Roehe et al., 2010; Dufrasne et al., 2013), suggesting that it is substantially affected by own (fetal) genetic factors as well as maternal genetic effects. Therefore, it is a worthwhile endeavor to determine which genes or variants underly this variation in birth weight.

A few birth weight related markers have been identified by the study of candidate genes such as *MYOG*, *MSTN* and *DBH* (Te Pas et al., 1999; Jiang et al., 2002; Tomás et al., 2006). With the widespread use of customized single nucleotide polymorphism (SNP) arrays, an increasing number of potential markers have been identified by genome-wide association study (GWAS). Wang X. et al. (2016) found over two hundred SNPs associated with birth weight by using first parity sows whose offspring had extreme birth weights; Zhang et al. (2018) identified 17 genomic regions associated with birth weight; Wang et al. (2017) found 12 SNPs that were significantly associated with piglet uniformity; and 27 differentially selected regions associated with the birth weight of piglets were detected by Zhang et al. (2014). However, a birth weight GWAS of Large white pigs by Wang et al. (2018) was unable to determine any significant loci. The identification of birth weight associated markers remains difficult to reproduce.

With rapid development of next-generation sequencing technology, a number of techniques have been widely adopted for genotyping, including whole genome resequencing and reduced-representation sequencing (RRS) techniques such as genotyping-by-sequencing and restriction site-associated DNA sequencing (RAD-seq) (Baird et al., 2008; Huang et al., 2009; Elshire et al., 2011). Compared to SNP chip analysis, RRS approaches are based on restriction site associated fragments and have great advantages in both the number of SNPs acquired and the ability to identify novel SNPs. Currently, RRS approaches are widely employed in combination with GWAS (Bhatia et al., 2013). As SNP chip analyses only share a small subset of SNPs with RRS (Brouard et al., 2017), the combination of the two methods in one population may improve repeatability of GWAS findings.

Trait related loci can be identified with GWAS, however, the elucidation of the causative variant rather than the loci is the ultimate goal. The determination of the causative variant requires a high density of SNPs in a particular region of GWAS. If the region is not genotyped at a sequence level, the imputation technique can be used to fill in missing SNPs from the available reference panels. Due to linkage disequilibrium between SNPs, the GWAS signal extends across a large region. Although it is not always possible to directly identify the causative variant, the region containing the causative variant can be narrowed down by sophisticated methods (Fang and Georges, 2016; Huang et al., 2017). The key feature of these methods is determining SNPs that have a 95% probability of containing the causative variants, as calculated with posterior probabilities.

In this study, we used the DNA variants from two different genotyping approaches, SNP chip and RAD-seq, to perform GWAS for birth weight. To finely map causative genes, we built a reference panel for a region-of-interest by deep resequencing of 28 boars, by which the merged SNPs of RAD-seq and SNP chip were imputed at the sequence level. Finally, we detected the potential causative genes within or close to the finely mapped region.

## Materials and Methods

### Animals and Phenotypes

Pedigree and phenotype records used for this study were provided by our lab. The pedigree contains 26,539 animals from 7 generations, including 14,226 Yorkshire and 12,313 Landrace animals. There were 12,661 and 10,635 records of birth weight for Yorkshire and Landrace piglets, respectively. After excluding disqualified records (missing birth date or abnormal records), 10,267 and 8,919 records Yorkshire and Landrace piglets were included, respectively. A total of 674 purebred sows (453 Yorkshire, 221 Landrace) born between 2014 and 2016 were selected for RAD-seq. After eliminating abnormal values (deviated from the third quartile), 668 high quality records were analyzed.

### RAD-seq With BGI-seq500

Genomic DNA was isolated from the ear tissue of pigs; the double-digest restriction enzyme associated DNA sequencing method (RAD-seq) was performed using the methods of Andolfatto et al. (2011) with appropriate modifications. Briefly, the DNA concentration of all samples was normalized to 50 ng/pr in 96-well plates, and digested with FastDigestTaq I- *Msp*I (Thermo Fisher Scientific) in 30 μL volume containing 20 μL DNA (1 μg). An anneal adapter (10 μM) was ligated to the digestion products by T4 DNA ligase with 23 *Taq*I-Ms. Then, 24 ligation products were pooled together to form one library with 15 μL per sample. Agencourt^®^ AMPure^®^ XP Reagent was used for library size-selection. The PCR system contained 50 ng size-selection products, 25 μL KAPA HiFi HotStart ReadyMix (kapasystem), and 10 pmol primers. PCR products were purified by Agencourt^®^ AMPure^®^ XP Reagent. The final library quality (concentration and fragment size distribution) was determined by a Qubit 2.0 Fluorometer (Thermo Fisher Scientific) and BiopticQsep100 DNA Fragment Analyzer (Bioptic), respectively. Every four library products (96 different barcodes) were mixed together in equal parts which a total weight at 170 ng. The cycling system contained 48 μL library mix, 1 × T4 DNA ligase buffer, 0.5 μL T4 DNA ligase (600 U/μL), and 100 pmol Splint Oligo, were reactions at 37°C and fragment size distribution were determined by a Qubit 2.0 Fluorometer (Thermo Fisher Scientific) and Bioptic Qsep100 DNA Fragment Analyze sample volume of Agencourt^®^ AMPure^®^ XP Reagent. Finally, the purified cyclizing libraries were sequenced with a BGI-seq500 platform (PE100).

Sequenced paired-end reads for each sow were identified by barcode and aligned against the *Sscrofa* reference genome (version *Sscrofa* 11.1)^1^ using the Burrows-Wheeler Aligner (version 0.7.12) software (Li and Durbin, 2009). SAMtools (version 0.1.19) (Li et al., 2009) was used to generate the consensus sequence for each sow and prepare input data for SNP calling with the Genome Analysis ToolKit (version 3.4) (McKenna et al., 2010). Raw SNPs with sequencing depth greater than 2,500 or less than 50 were removed, as SNP with extreme sequencing depth is most likely caused by a repeat DNA sequence or alignment error. The SNPs underwent quality control (QC) in which those with a call rate > 0.5, minor allele frequency (MAF) > 0.05, and *p*-value > 10^–6^ for the Hardy-Weinberg equilibrium test were kept, resulting in 140,948 SNPs. The missing genotypes were imputed with Beagle software (Browning and Browning, 2007), and the SNPs were filtered again with the above QC criteria. Finally, 139,634 high quality SNPs were retained for subsequent analysis.

### SNP Chip Genotyping

These individuals were also genotyped with a Geneseek Porcine 50K SNP Chip (Neogen, Lincoln, NE, United States), which contained 50,697 SNPs across autosomes and sex chromosomes. QC of the SNPs was conducted using PLINK (version 1.07) (Purcell et al., 2007). The SNPs with MAF > 0.05, call rate > 0.97, and individual call rate > 0.95 were retained. Furthermore, we removed SNPs that were not mapped to the *Sscrofa* 11.1 genome, leaving 45,180 SNPs. The missing genotypes were imputed with Beagle software and underwent QC with the above QC criteria. Finally, 45,175 high quality SNPs were included.

### Whole Genome Sequencing

We sequenced the whole genome of 28 boars, the ancestors of 453 Yorkshire sows (unpublished), with an average sequence depth ∼19× (ranged from 17.06× to 22.24×). After genome alignment with Burrows-Wheeler Aligner and SNP calling with the Genome Analysis Toolkit, 17,017,067 raw SNPs were detected. These SNPs were filtered using the Genome Analysis Toolkit with parameters “QUAL < 30 || QD < 2.0 || FS > 60.0 || MQ < 40.0 || MQRankSum < −12.5 || ReadPosRankSum < −8.0,” and using PLINK with MAF < 0.05 and *p*-value < 10^–6^ for the Hardy-Weinberg equilibrium test. We removed 761,590 additional SNPs with missing genotypes across the 28 boars, leaving 11,668,346 high quality SNPs, which were taken as the reference panel for imputation.

### Sequence Level Imputation

The SNPs determined by RAD-seq (140,948 SNPs) and SNP chip (45,180 SNPs) were merged to produce a high density SNP set for sequence level imputation. After removing 427 duplicate SNPs from both SNP sets, 185,701 SNPs remained. We performed sequence level imputation with Beagle by taking the whole genome sequencing data of 28 Yorkshire boars (described above) and 20 Landrace pigs (downloaded from https://figshare.com/articles/data2019_tar_gz/9505259). After QC (MAF < 0.05 and *p*-value < 10^–6^), we obtained 9,012,073 overlapping SNP markers for the two breeds and imputed the RAD_ chip SNPs of the Yorkshire and Landrace pigs to a genome-wide level.

### Variance Component Estimation and Heritability

Both pedigree and RAD_SNP information were used to build a kinship matrix among individuals to estimate the variance components of birth weight. The mixed linear model for this estimation was:

Y=X⁢b+Z⁢u1+Z⁢p2+e

where Y is the phenotype vector, b is a fixed effects vector, i.e., herd-year-season, sex (only in pedigree-based estimation), breed (2 breeds in SNP-based and 6 strains in pedigree-based estimation) and birth parity, u is a vector of additive genetic effects following the multinormal distribution: u ∼ N (0, A$\sigma_{a}^{2}$) and ∼ N (0, G$\sigma_{a}^{2}$), respectively in pedigree and RAD_SNP based estimations, where A is the pedigree relationship matrix and G is the genomic relationship matrix constructed based on SNPs as described in VanRaden (2008). p is a material effects vector: p ∼ N (0, I$\sigma_{p}^{2}$) and e is a residuals vector: e ∼ N (0, I$\sigma_{e}^{2}$), and I is an identity matrix. $\sigma_{a}^{2}$, $\sigma_{p}^{2}$, and $\sigma_{e}^{2}$ are the additive genetic, material genetic, and residual variances, respectively. X, Z_1_, and Z_2_ are the incidence matrices for b, u, and p, respectively. The variance components were estimated using the average information restricted maximum likelihood procedure in DMU software (version 6, release 5.2^2^). Heritability of birth weight was estimated as:

h=2σa2σp2+σa2+σe2

The standard error of heritability was obtained as Klei and Tsuruta (2008) described.

### Genome-Wide Association Study

The mixed model including a random polygenic effect can be expressed as:

Y=X⁢b+Z⁢a+M⁢g+e

where Y is the phenotype vector, which is corrected with estimated breeding values and fixed effects (only residuals left), and estimated breeding values are evaluated with the average information restricted maximum likelihood procedure in DMU; b is the estimator of fixed effects including breed, g is the SNP substitution effect and a is the vector of random additive genetic effects following the multinormal distribution a ∼ N (0, G$\sigma_{a}^{2}$), in which G is the genomic relationship matrix that is constructed based on SNPs as described in VanRaden (2008), and $\sigma_{a}^{2}$ is the polygenetic additive variance. X, Z, and M are the incidence matrices for b, a, and g, respectively. e is a vector of residual errors with a distribution of N (0, I$\sigma_{e}^{2}$). All single-marker GWAS analyses were conducted using the EMMAX software (Kang et al., 2010). Based on the Bonferroni correction, the genome-wide significant threshold was P < 1/N, where N is the number of informative SNPs.

### Fine-Mapping

The BayesFM-MCMC package (Fang and Georges, 2016) was used to finely map causative variants, in which the threshold for SNP clustering was set as *r*^2^ = 0.5; the length of the Markov chain was 510,000 with the first 10,000 discarded (burn-in period). The threshold to declare significance was set at 1.1 × 10^–5^, which was determined from 0.05 divided by the number of SNPs in the GWAS region. We corrected the phenotypes by subtracting the corresponding breeding values and fixed effects, where the breeding values were estimated via the DMU package.

### Gene-Annotation

SnpEff (version 4.3t) (Cingolani et al., 2012) was used to annotate the function of SNPs, in which the genome sequence and the genomic annotation databases (.gff) were required. The Sscrofa11.1 genome were downloaded from the National Center for Biotechnology Information^3^ and the genomic annotation file (.gff) was downloaded from the web ftp://ftp.ncbi.nlm.nih.gov/genomes/all/GCF/000/003/025/GCF_000003025.6_Sscrofa11.1/GCF_000003025.6_Sscrofa11.1_genomic.gff.gz.

## Results

### RAD-seq and SNP Chip Genotyping

**FIGURE 1:**
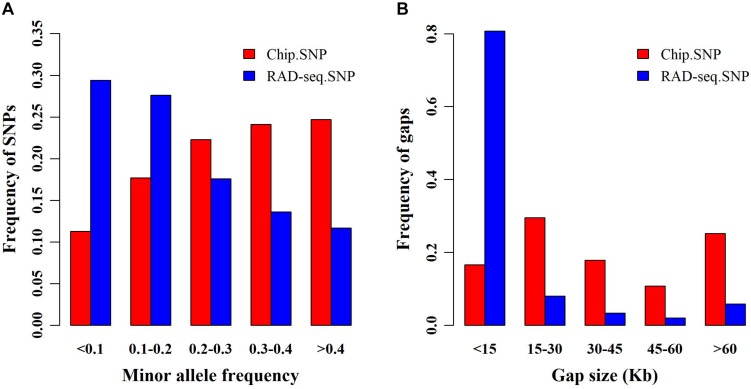
Minor allele frequency (MAF) and distance between single nucleotide polymorphisms (SNPs). **(A)** Frequency of SNPs in different MAF classes for restriction site associated DNA sequencing (RAD-seq) and SNP chip assays. **(B)** Frequency distribution of the distance between adjacent SNPs for RAD-seq and SNP chip assays.

We obtained 139,634 SNPs from RAD-seq and only 45,175 SNPs from SNP chip analysis. First, we compared the allele frequencies (AF) of SNPs garnered from both genotyping platforms (Figure 1A). Compared with SNP chip analysis, RAD-seq more frequently found SNPs with lower AF. Specifically, the likelihood of RAD-seq finding SNPs with AF < 0.1 was nearly 0.3, almost two times higher than that of SNP chip analysis (∼0.1). We also compared the distance between adjacent SNPs determined by the two genotyping methods (Figure 1B). The adjacent SNPs found by RAD-seq were much closer together than those found with SNP chip analysis, suggesting that RAD-seq is more informative and may be helpful to detect causative genes. Finally, we determined the overlapping SNPs between the two SNP sets, and surprisingly found only 427 SNP overlaps.

### Genome-Wide Association Study

We estimated heritability prior to the association study to fully understand how much birth weight is inherited. We used pedigree information and genome SNPs to estimate heritability. There were 14,226 and 12,313 individuals in the pedigree, and 10,267 and 8,919 records of birth weight for Yorkshire and Landrace, respectively. Genome-wide SNP information was used to build kinship among individuals and heritability was estimated as 0.094 ± 0.065. Then, once again using the pedigree, we estimated heritability in Yorkshire and Landrace pigs at 0.162 ± 0.026 and 0.131 ± 0.025, respectively (see Table 1), which are closer to previous reports than the heritability found when genome-wide SNP information was used.

**TABLE 1:** The estimated heritability of birth weight for Yorkshire and Landrace with different sources of information.

**Population**	**Information**	**Number of pigs**	**Number of pigs**	**$\sigma_{a}^{2}$**	**$\sigma_{p}^{2}$**	**$\sigma_{e}^{2}$**	**h^2^**
	**sources**	**in pedigree**	**with BW records**				
Yorkshire	Pedigree	14,226	10,267	0.011 (0.002)	0.013 (0.001)	0.043 (0.001)	0.162 (0.026)
Landrace		12,313	8,919	0.011 (0.002)	0.017 (0.001)	0.056 (0.002)	0.131 (0.025)
Yorkshire & Landrace	SNPs	–	668	0.007 (0.005)	0.024 (0.005)	0.042 (0.005)	0.094 (0.065)

*$\sigma_{a}^{2}$, $\sigma_{p}^{2}$, and $\sigma_{e}^{2}$ are the additive genetic variance, material genetic variance and residual variance, respectively; standard errors are in parentheses.*

**FIGURE 2:**
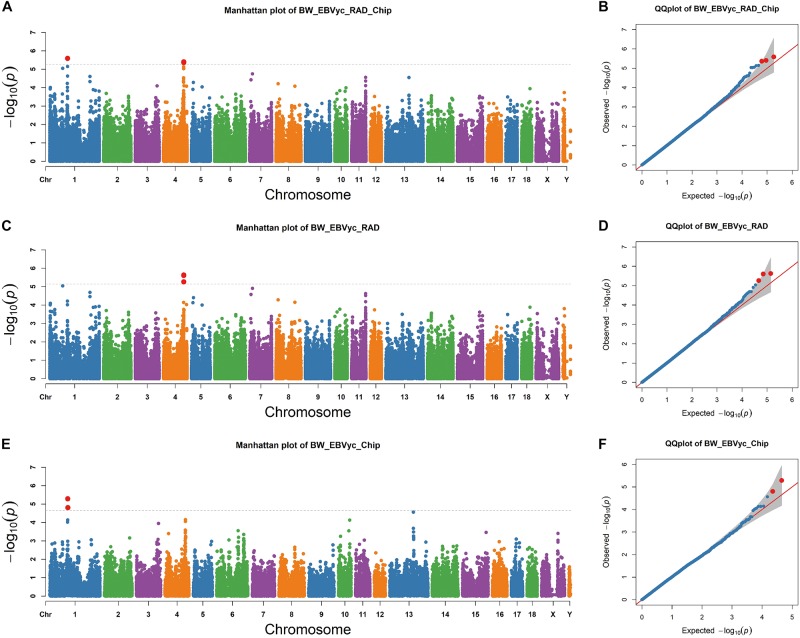
Genome-wide association study (GWAS) profiles from the merged SNPs of RAD-seq and SNP chip assays **(A,C,E)** and the corresponding Q-Q plot **(B,D,F)** the horizon lines represent the thresholds as determined by Bonferroni correction.

Next, we performed an association study for genome-wide SNPs based on a mixed model that accounted for population kinship (see section “Materials and Methods”). SNP sets from RAD-seq and SNP chip analysis were merged together, with two signals on chromosome 1 and 4 exceeding the threshold (Figure 2A). The positions of the lead SNPs for the two regions were chr1: 97,745,041 and chr4: 112,031,589, respectively; the MAF of the lead SNPs were 0.24 and 0.34 and they explained 6.36% and 4.25% of the phenotypic variance, respectively. We then focused on the two GWAS regions surrounding the lead SNPs, which are determined as the surrounding 1∼2 Mb region around the lead SNP. To confirm the two GWAS signals, we performed separate GWAS for the RAD-seq and SNP chip datasets. The region on chromosome 4 was determined to be significant for the RAD-seq dataset but not for the SNP chip dataset; where the reverse was true for the region on chromosome 1 (Figures 2C,E). Despite only reaching significance in one dataset, the –logP values of both regions peak in both datasets, confirming the reliability of the GWAS signals. To check for false positives caused by population stratification, we closely examined the theoretical and observed *p*-values with a Q-Q plot^4^. The -logP values are well fit by a linear regression against theoretical -logP values (Figures 2B,D,F), suggesting that population stratification has been well corrected for, although, it is important to note that two breed populations were simultaneously investigated.

### Fine Mapping

To further refine the regions containing causative genes and variants, we performed fine mapping of the GWAS region 1∼2 Mb around the lead SNP. To increase fine mapping accuracy, we utilized as many SNPs as possible by merging the SNPs from both RAD-seq and SNP chip analysis and removing duplicate SNPs. After applying a stringent filter, we obtained 5,226 and 7,184 SNPs in the fine mapping regions of chromosome 1 and 4, respectively. With this high density of SNPs, we were able to impute SNPs at a sequence level. Sequence-level imputation requires a sequence-level reference SNP set. We therefore re-sequenced 28 Yorkshire boars with an average coverage of ∼19x and downloaded the whole genome sequencing data of 20 Landrace pigs. This resulted in 11,668,346 and 18,954,748 sequence-level SNPs for Yorkshire and Landrace pigs, respectively. With these SNPs as a reference panel, we imputed the merged RAD-seq and SNP chip SNPs at a sequence level using Beagle software separately for each breed. Then, we employed BayesFM-MCMC software to narrow down the clusters containing causative variants. BayesFM-MCMC first clusters the SNPs within a GWAS region using a hierarchy clustering algorithm according to *r*^2^ among SNPs; then it models multiple causal variants by carrying out a Bayesian model selection across the cluster and generates the posterior probability for each SNP within the cluster, by which a credible set of SNPs with >95% posterior probability is constructed. The advantages of BayesFM-MCMC are that (1) it narrows down potential causative variants by indicating causal variants in the SNP set; and (2) it efficiently identifies more than one variant if multiple variants control the investigated trait.

**FIGURE 3:**
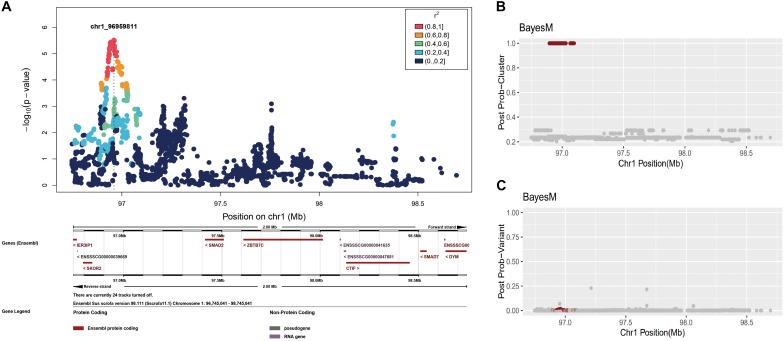
Fine-mapping in the chromosome 1: 96,745,041–98,745,041 region. **(A)** Individual SNP association study and its locuszoom plot. **(B)** The posterior probability of clusters. **(C)** The posterior probability of SNPs.

However, because BayesFM-MCMC does not solve a mixed model with polygenic effects, we corrected the phenotype values by using the residuals (see section “Materials and Methods”). First, we conducted a single variant association for the GWAS chromosome region, 1,96,745,041–98,745,041, which produced a sharp peak in this region (Figure 3A). We then employed BayesFM-MCMC to further refine the regions, and one cluster signal with *a posterior* probability equal to 1 (greater than the threshold 0.5) was identified. To examine which SNPs predominantly explained the posterior probability in this cluster, we plotted the posterior probabilities for each SNP (output from BayesFM-MCMC). Most SNPs have miniscule posterior probabilities and no one SNP gives substantial posterior probability (f.i. greater than 0.5 or 0.2) in the identified cluster (Figures 3B,C). We then employed the 95% credible set defined by BayesFM-MCMC to further refine the causal variants, which contained 71 SNPs across a ∼100 kb region from 96,895,307 to 97,098,059 (see Supplementary Table S1 for detail). This 100 kb region contained the peak identified with the scan of single variants (Figure 3A), confirming the refined 100 kb region was reliable.

**FIGURE 4:**
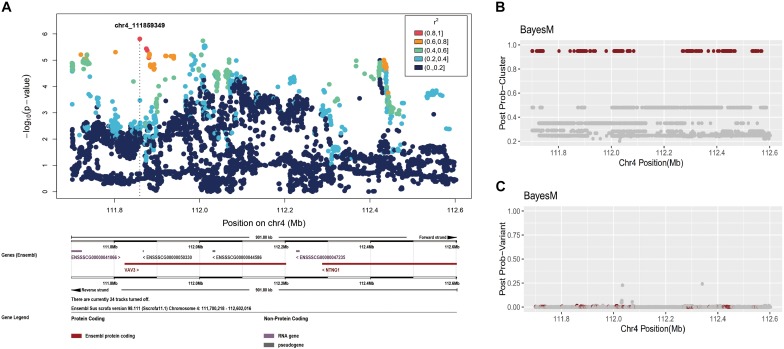
Fine-mapping in the chromosome 4: 111,031,589–113,031,589 region. **(A)** Individual SNP association study and its locuszoom plot. **(B)** The posterior probability of clusters. **(C)** The posterior probability of SNPs.

Fine mapping of the region on chromosome 4, 111,031,589–113,031,589 (Figure 4B), identified one cluster signal with *a posterior* probability equal to 1. As before, we plotted the posterior probabilities for each SNP but most SNPs once again had miniscule posterior probabilities (less than or 0.05) (Figure 4C). The 95% credible set of causal variants in chromosome 4 contained 432 SNPs across over a ∼870 kb region from 111,700,218 to 112,569,735 (see Supplementary Table S2 for detail). The peak found in the single-SNP association profile (Figure 4A) is covered by this ∼870 kb region, once again confirming the reliability of BayesFM-MCMC for this purpose. The correlation (*r*^2^) among SNPs confirmed that they were highly linked, which explains why the individual posterior probabilities of these SNPs are very small.

### Candidate Genes

The 71 SNPs of interest on chromosome 1 are located in the intergenic region, which lies about 53 kb upstream of *SKOR2* and over 317 kb downstream of *SMAD2* (Table 2, see Supplementary Table S1 for details). We hypothesize that these variants are likely to have regulatory effects on the two nearby genes.

**TABLE 2:** Candidate genes for birth weight located around the causal variants.

**chr**	**hgnc_symbol**	**Start_position**	**End_position**	**Function**	**Reported in pig and other species**
chr4	VAV3	111825071	112202833	Guanine nucleotide exchange factors (GEFs) for Rho family GTPase	Pig food conversion ratio (Wang et al., 2015)
	NTNG1	112280899	112615782	Guides axon growth during neuronal development	Calf birth weight (Cole et al., 2014)
	SLC25A24	111580186	111632390	Calcium-dependent mitochondrial solute carrier	Bovine embryonic mortality (Killeen et al., 2016)
	PRMT6	112698865	112710305	Mediates the asymmetric dimethylation of Arg2 of histone H3	Bulls sperm concentration (Hering et al., 2014)
	STXBP3	111234154	111294067	Insulin-regulated *GLUT4* trafficking	Be positively selected for body weight (Li et al., 2014)
chr1	SKOR2	96795507	96842064	Negatively regulate TGFβ signaling pathways	More rapid weight gain in African American males (Tu et al., 2015)
	SMAD2	97415716	97,511,358	Mediates the signal of the transforming growth factor (TGF)-beta	Causative gene for dog body size (Rimbault et al., 2013)

The 432 highly linked SNPs on chromosome 4 are located within four genes, *LOC106510205* (covered by 28 SNPs), *LOC106510207* (covered by 26 SNPs), *VAV3* (covered by 160 SNPs), and *NTNG1* (covered by 218 SNPs, see Supplementary Table S2). Among these SNPs, one is a coding amino acid, seven are located in the 3′ untranslated region and 414 are located in the intron (see Supplementary Table S2 for details). The coding variant is a synonymous variant (c.1136 T > A), localized in gene *VAV3*. The remaining variants are in non-coding sites distributed in all four genes, suggesting the causal variant may have regulatory effect. We searched for functional genes near the tightly linked region, and thereby included *SLC25A24*, *PRMT6*, *STXBP3* as candidate genes (Table 2).

## Discussion

We employed two genotyping methods, RAD-seq and a customized SNP chip assay, to obtain genome-wide distributed SNPs. The number of SNPs identified by RAD-seq was three times greater than those identified by customized SNP chip, among these, only 427 SNPs overlapped, consistent with previous reports (Brouard et al., 2017). Furthermore, we found that RAD-seq was able to genotype more low-frequency SNPs than the SNP chip assay. As we known, rare and low frequency variants have been found to partially explain phenotypic variation in some human diseases and agricultural traits (Quintana-Murci, 2016; Zhang et al., 2017).

By using genome-wide association combined with post-GWAS fine mapping, we refined one causative variant to a ∼100 kb region containing 71 SNPs. This region is located in the intergenic region between *SKOR2* and *SMAD2*. Intergenic sequences are generally considered as junk sequences. However, in recent years, studies have increasingly shown that intergenic sequences contain long-distance regulatory elements and may also generate a large amount of non-coding RNA through transcription, thereby regulating the expression of surrounding genes (Chen and Tian, 2016). *SKOR2* is homologous to the Ski/Sno family of transcriptional co-repressors, which has been shown to negatively regulate transforming growth factor β (TGFβ) signaling pathways by binding to Smads (Arndt et al., 2005). *SKOR2* null mice are smaller than their siblings (Wang W. et al., 2011). *SKOR2* polymorphism has also been reported to be associated with more rapid weight gain in African American males (Tu et al., 2015). *SMAD2* is activated by TGFβ, and regulates multiple cellular processes, such as cell proliferation, apoptosis, and differentiation. As we known, TGFβ pathways play critical roles in bone development (Li et al., 2005). *SMAD2* plays an essential role in regulating chondrocyte proliferation and differentiation in the growth plate (Wang W. et al., 2016). Additionally, *SMAD2* was identified as the causative gene for the body-size of dogs, and was associated with the total number of piglets born in Yorkshire pigs as well as with high fecundity in dairy goats (Rimbault et al., 2013; Lai et al., 2016; Wang et al., 2018). Our results suggest that causative variants in this intergenic region may contribute to birth weight phenotypes by interfering with the regulatory function of the nearby distal regulatory elements and causing differential expression of the two surrounding genes.

We have refined the causative variant on chromosome 4 to a ∼870 kb region which resides in a big linkage disequilibrium block containing 4 genes, *LOC106510205*, *LOC106510207*, *VAV3*, and *NTNG1. NTNG1* plays an important role in cell signaling during nervous system development (Nakashiba et al., 2000) and is associated with calf birth weight in Holstein cattle (Cole et al., 2014). *LOC106510205* and *LOC106510207* are predicted to be long non-coding RNA (lncRNA), and has not been functionally characterized to this point. As we known, lncRNA transcription plays an important role in both *cis*- and trans-regulation of nearby gene expression (Sun and Kraus, 2015). *VAV3* is located in the center of the fine mapping region and is near the two lncRNAs. *VAV3* is a member of the *VAV* gene family that activates actin cytoskeletal rearrangement pathways and transcriptional alterations (Zeng et al., 2000). *VAV3* is versatile and also regulates osteoclast function, bone mass, and the homeostasis of the cardiovascular and renal systems (Faccio et al., 2005; Sauzeau et al., 2006). Previous knock-out results have shown that *Vav3*-deficient mice were protected from bone loss induced by systemic bone resorption stimuli such as parathyroid hormone or RANKL (Faccio et al., 2005). Furthermore, *VAV3* is associated with hypothyroidism in humans, food conversion ratio in a male Duroc pig population, high body weight and growth rate in Boer goats, as well as sperm concentration in Holstein-Friesian bulls (Hering et al., 2014; Kwak et al., 2014; Wang et al., 2015; Onzima et al., 2018).

Several genes near the ∼870 kb tightly linked region were found to be related to growth and development or have been identified in others studies (Table 2). For example, *SLC25A24* encodes a carrier protein that mediates electroneutral exchange of Mg-ATP or Mg-ADP against phosphate ions, is responsible for low fat mass in humans and mice (Urano et al., 2015), and is also related with bovine embryonic mortality (Killeen et al., 2016). Mutations in *SLC25A24* have been found to be associated with fontaine progeroid syndrome in humans (Rodríguez-García et al., 2018). Furthermore, *STXBP3* (also known as *Munc18c*), involved in insulin-regulated *GLUT4* trafficking, has been found to be positively associated with body weight in Large White and Tongcheng pigs (Li et al., 2014). Finally, *PRMT6*, is reported to be associated with bull sperm concentration (Hering et al., 2014), and the expression of *PRMT6* in skeletal muscle has been found to be regulated with a strong *cis*-expression quantitative trait loci (personal communication). Taken together, the region spanning *VAV3* and *NTNG1* is a very important genetic factor underlying the birth weight of pigs.

Most of the finely mapped SNPs obtained herein were located in intergenic regions or within introns. Therefore, we propose that these variants may have a regulatory effect on the expression of nearby genes, such as *SKOR2*, *SMAD2*, *VAV3*, and *NTNG1*, and thereby regulating body development. This research did not confirm such regulatory mechanisms but has highlighted them for further investigation.

## Conclusion

We used the DNA markers from two different genotyping approaches to perform GWAS, and identified significant loci in chromosome 1 and chromosome 4 which explained 6.36% and 4.25% of the phenotypic variance, respectively. To increase the accuracy of fine mapping, we imputed the merged RAD-seq and SNP chip SNPs at a sequence level using the SNPs of high-coverage resequenced pigs as a reference panel. Then, we employed BayesFM-MCMC software to narrow down the genomic region of the clusters that contained causative variants. One cluster was located in an intergenic region, and the other in a gene coding region. Finally, we identified four promising candidate genes, *SKOR2*, *SMAD2, VAV3, NTNG1*, that have been associated with growth related traits in other species including cattle, humans, and dogs. Most SNPs in the fine mapping region were located in the intergenic region or introns, and as such we propose that these variants may have a regulatory effect on the expression of nearby genes, which deserves further investigation. The birth weight of pigs is an important economic factor in the livestock industry, identification of a causal variant would be beneficial to the molecular breeding of pigs.

## Data Availability Statement

The datasets generated for this study can be found in the https://figshare.com/articles/GWAS_datasets/9917462.

## Ethics Statement

This study was carried out in accordance with the guidelines of the Science Ethics Committee of the Huazhong Agricultural University (HZAU). All animal experiments were approved by the Institutional Review Board on Bioethics and Biosafety of Beijing Genomics Institute (BGI-IRB).

## Author Contributions

SZ, MF, and YL conceived and designed the experiments. YL, MY, RW, QW, LY, XL, and XX performed the experiments. YL, BL, MF, TC, HH, ZM, and JS analyzed the data. YL, MF, and BL wrote the manuscript.

## Conflict of Interest

The authors declare that the research was conducted in the absence of any commercial or financial relationships that could be construed as a potential conflict of interest.

## Footnotes

1 https://www.ncbi.nlm.nih.gov/genome/84?genome_assembly_id=317145

2 http://dmu.agrsci.dk/

3 https://www.ncbi.nlm.nih.gov/genome/84?genome_assembly_id=317145

4 https://github.com/YinLiLin/R-CMplot

## Supplementary Material

TABLE S1 Gene annotation of 71 single nucleotide polymorphisms in the fine mapping region of chromosome 1.xlsx.

TABLE S2 Gene annotation of 432 single nucleotide polymorphisms in the fine mapping region of chromosome 4.xlsx.
